# Passive Acoustic Source Localization at a Low Sampling Rate Based on a Five-Element Cross Microphone Array

**DOI:** 10.3390/s150613326

**Published:** 2015-06-05

**Authors:** Yue Kan, Pengfei Wang, Fusheng Zha, Mantian Li, Wa Gao, Baoyu Song

**Affiliations:** 1 State Key Laboratory of Robotics and System, Harbin Institute of Technology, Harbin 150001, China; E-Mails: kyh_7372@163.com (Y.K.); wangpengfei1007@163.com (P.W.); limt@hit.edu.cn (M.L.); skymoon.hit@gmail.com (W.G.); 2 Department of Mechanical Design, Harbin Institute of Technology, Harbin 150001, China; E-Mail: sby@hit.edu.cn

**Keywords:** passive acoustic source localization, time delay of arrival (TDOA), generalized cross-correlation (GCC), up-sampling (US), interpolation factor, five-element cross microphone array

## Abstract

Accurate acoustic source localization at a low sampling rate (less than 10 kHz) is still a challenging problem for small portable systems, especially for a multitasking micro-embedded system. A modification of the generalized cross-correlation (GCC) method with the up-sampling (US) theory is proposed and defined as the US-GCC method, which can improve the accuracy of the time delay of arrival (TDOA) and source location at a low sampling rate. In this work, through the US operation, an input signal with a certain sampling rate can be converted into another signal with a higher frequency. Furthermore, the optimal interpolation factor for the US operation is derived according to localization computation time and the standard deviation (SD) of target location estimations. On the one hand, simulation results show that absolute errors of the source locations based on the US-GCC method with an interpolation factor of 15 are approximately from 1/15- to 1/12-times those based on the GCC method, when the initial same sampling rates of both methods are 8 kHz. On the other hand, a simple and small portable passive acoustic source localization platform composed of a five-element cross microphone array has been designed and set up in this paper. The experiments on the established platform, which accurately locates a three-dimensional (3D) near-field target at a low sampling rate demonstrate that the proposed method is workable.

## 1. Introduction

Passive acoustic source localization has been extensively investigated in the last two decades. Time delay of arrival (TDOA)-based methods are widely used in this area for their simple implementation and small computational complexity [1]. Firstly, TDOA-based methods estimate the time delay between two spatially-distributed microphones. Secondly, acoustic source location is derived from the corresponding non-linear localization equations according to TDOA estimations and the microphone array geometric position. An accurate time delay estimation (TDE) is essential for the good performance of the acoustic source location based on the TDOA method, since any error in the TDE leads to a high error of the target location estimation [2]. In general, the generalized cross-correlation (GCC) method is applied to estimate the TDOA. In practice, the performance of time delay estimation based on the GCC method is dependent on the sampling rate [3], namely a high sampling rate [4–6] can contribute to a high localization accuracy. However, some localization systems (e.g., wearable localization systems, hearing aid and human-computer interactions) tend to be small and portable with the development of the integrated circuit, electronic and computer technology, *etc*. For theses portable systems and micro-embedded systems [7,8] it is challenging to improve the localization accuracy by increasing the sampling rate, because of the limitation of the system size, hardware and power consumption, *etc*. Hence, it is important that sampling rate conversion (SRC) be exploited to improve localization accuracy under a low sampling rate. There is the up-sampling (US) method in the SRC field that can increase the original sampling rate of the input signal. In this sense, to achieve accurate localization at a low sampling rate, a modification of the GCC method is proposed based on the US theory and defined as the US-GCC. The US theory can be used to complete an interpolation processing in regards to the signals sampled under the low sampling rate. The TDOA between the output signals through the interpolation processing is then estimated by the GCC method. In addition, the reasonable interpolation factor is the crucial problem for the US theory. Increasing the interpolation factor can result in the increase of the sampling rate, as well as the improvement of the TDOA estimation and localization accuracy. Meanwhile, localization computation time and storage space will increase with the increase of the interpolation factor. Therefore, for the near-field acoustic source in this paper, the optimal interpolation factor is selected according to localization computation time and the standard deviation (SD) of target location estimation.

The remainder of this paper is organized as follows. In Section 2, a new localization algorithm is described under the low sampling rate based on GCC method and US theory. Meanwhile, the optimal interpolation factor is discussed and selected for the US operation. In Section 3, for evaluation purposes, localization error comparison based the GCC method and the proposed method is presented by simulation. Furthermore, the established simple portable passive acoustic source localization platform can complete accurate acoustic source localization at a low sampling rate. In Section 4, the paper is completed with some concluding remarks.

## 2. Methods

### 2.1. Proposed Localization Algorithm Based on the Generalized Cross-Correlation Method and Up-Sampling Theory

Generally, the TDE of one pair of microphones has been acquired using the GCC method [9]. Considering *x*_1_(*n*) and *x*_2_(*n*) as the received signals at Microphone 1 and Microphone 2, the cross-correlation function between *x*_1_(*n*) and *x*_2_(*n*) is written as:

(1) $$\begin{array}{cc} R_{x_{1}x_{2}}\left(\tau \right) & =E\left[x_{1}\left(n\right)x_{2}\left(n-\tau \right)\right]\\ & =R_{x_{1}x_{2}}\left[\tau -\left(\tau_{1}-\tau_{2}\right)\right] \end{array}$$

where *τ_i_* (*i* = 1, 2) is the propagation time from the acoustic source to the microphones and *τ*_1_ − *τ*_2_ is the time delay between the signals arriving at Microphone 1 and Microphone 2. According to the characteristics of the cross-correlation function, *R*_*x*_1___*x*_2__ (*τ*) ideally should exhibit a prominent peak when *τ* = *τ*_1_ − *τ*_2_. That is, the time delay estimation *τ_m_* is obtained via maximizing the cross-correlation function defined by Equation (1):

(2) $$\tau_{m}=\tau |R_{x_{1}x_{2}}\left(\tau \right)\;\text{is}\max$$

In practice, to sharpen the cross-correlation function peak and limit the impact caused by noise and reverberation, the cross-correlation function is transformed into the cross-correlation spectrum function through Fourier transform. Then, the weighting function is employed for the cross-correlation spectrum function. Finally, through Fourier inverse transform of the weighted cross-correlation spectrum function, the generalized cross-correlation function is defined as:

(3) $$R_{x_{1}x_{2}}^{G}\left(\tau \right)=\int_{-\infty }^{+\infty }\psi_{x_{1}x_{2}}\left(f\right)G_{x_{1}x_{2}}\left(f\right)e^{j2f\pi \tau }df$$

where *G*_*x*_1___*x*_2__(*f*) is the cross-correlation spectrum density function, Ψ_*x*_1___*x*_2__(*f*) is a weighting function and f is the frequency variable. For many different weighing functions, a commonly-used weighting function in acoustic event localization is the phase transform (PHAT), which is usually considered useful in reverberant conditions [10] and has low computational complexity and a higher recognition rate [3,4]. It can be described with the following equation:

(4) $$\psi_{x_{1}x_{2}}\left(f\right)=\frac{1}{\left|G_{x_{1}x_{2}}\left(f\right)\right|}$$

Inserting Equation (4) into Equation (3), the estimation of the TDOA for each microphone pair is computed as follows:

$$\tau_{\text{GCC}}=\tau |R_{x_{1}x_{2}}^{G}\left(\tau \right)\;\text{is}\max$$

However, due to the discretization of the input signal, the obtained TDE from Equation (5) must be converted into Equation (6):

(6) $$\begin{array}{cc} \tau_{ij} & =\Delta n_{ij}T\\ & =\frac{\Delta n_{ij}}{F} \end{array}$$

where *τ_ij_*, Δ*n_ij_*, *T* and *F* are TDOA estimation, sampling point, sampling period and sampling frequency, respectively.

It is clear that increasing the sampling frequency *F* results in the reduction of the error of the TDOA estimation *τ_ij_*. Yet, for a small portable system, especially for a multitasking micro-embedded system, to improve the localization accuracy depending on a high sampling rate is quite difficult because of the limitation of the system size, hardware and power consumption, *etc*. In the SRC field [11,12], US theory usually is used to increase the sampling rate of the input signal. Therefore, in order to complete the accurate localization at a low sampling rate, a modification of the GCC method based on the US theory is proposed and defined as the US-GCC method.

The US operation with a positive integer interpolation factor *L* is implemented by equidistantly inserting *L* − 1 zero-value sample points between two consecutive samples of the input signal, as shown in Equation (7):

(7) $$y\left(n\right)=\{\begin{array}{ll} x\left(\frac{n}{L}\right) & n=0,\pm L,\pm 2L,\cdots \\ 0 & \text{otherwise} \end{array}$$

where *x*(*n*) is the input signal and *y*(*n*) is the output signal through the US operation. The US develops *y*(*n*) with a sampling frequency that is *L*-times larger than that of *x*(*n*), namely:

(8) $$F_{y}=L\cdot F_{x}$$

where *F_x_* and *F_y_* are the sampling frequency of *x*(*n*) and *y*(*n*), respectively.

In addition, in terms of the z-transform, the input-output relation is then given by Equation (9):

(9) $$\begin{array}{ll} Y\left(z\right) & =\overset{\infty }{\underset{n=-\infty }{\text{∑}}}y\left(n\right)z^{-n}\\ & =\overset{\infty }{\underset{n=-\infty }{\text{∑}}}x\left(\frac{n}{L}\right)z^{-n}\\ & =X\left(z^{L}\right) \end{array}$$

By substituting *z*=*e^jω^* into Equation (9), the obtained *Y*(*e^jω^*)=*X*(*e^jωL^*) shows that the frequency spectrum of *y*(*n*) is *L*-times the repetition of the frequency spectrum of *x*(*n*) after the US operation. In addition, because of the *L*-times sampling rate expansion, there will be *L* − 1 additional images of the frequency spectrum of the input signal. Clearly, a low-pass filtering is employed to remove the *L* − 1 additional images.

**Figure 1:**
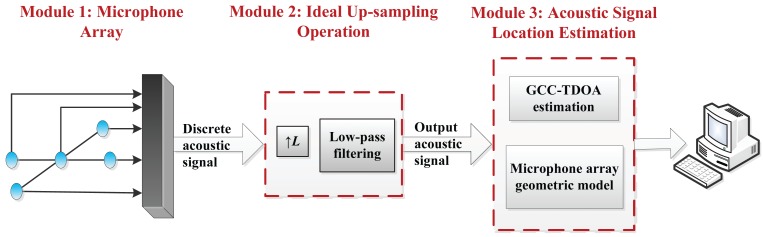
Block diagram of the GCC method for the time delay estimation (TDE) based on the US operation that is defined as the US-GCC method, where the US operation includes both part of the L-times interpolation and low-pass filtering.

Here, based on the US theory and the GCC method, the proposed localization algorithm under a low sampling rate is shown in Figure 1, and the process comprises the following steps.

Step 1: Through the US operation (interpolation factor *L*) for the collected discrete signal (a sampling rate of less than 10 kHz) from the microphone array, we will get the output signal with the higher sampling rate.
Step 2: TDOAs of the output signals through the processing in Step 1 are estimated by the GCC method.
Step 3: Acoustic signal location can be estimated according to the TDE in Step 2 and the microphone array geometric model.

### 2.2. Parameter Analysis

With reference to the US-GCC method, apparently the interpolation factor selection is crucial in order to effectively improve the localization accuracy. The interpolation factor is too small to effectively reduce the acoustic source location error, or it is too big that will it increase the calculation complexity and computation time. Then, the main results of the interpolation factor parameter analysis are given in the following theorem and inferences.

Theorem for the GCC method: The localization accuracy is relevant to the sampling rate, namely the high localization accuracy needs a high sampling rate.

Proof of the theorem: Firstly, the error of the *τ_ij_* is presented by Equation (10) taking into account a derivative with respect to *τ_ij_* of Equation (6) in Section 2.1.

(10) $$\begin{array}{ll} \delta \tau_{ij} & =\left(\frac{\partial \tau_{ij}}{\partial \Delta n_{ij}}\right)\delta \Delta n_{ij}+\frac{\partial \tau_{ij}}{\partial F}\delta F\\ & =\frac{F\delta \Delta n_{ij}-\Delta n_{ij}\delta F}{F^{2}} \end{array}$$

where *δτ_ij_, δ*Δ*n_ij_* and *δF* represent the error of *τ_ij_*, Δ*n_ij_* and *F*, respectively.

**Figure 2:**
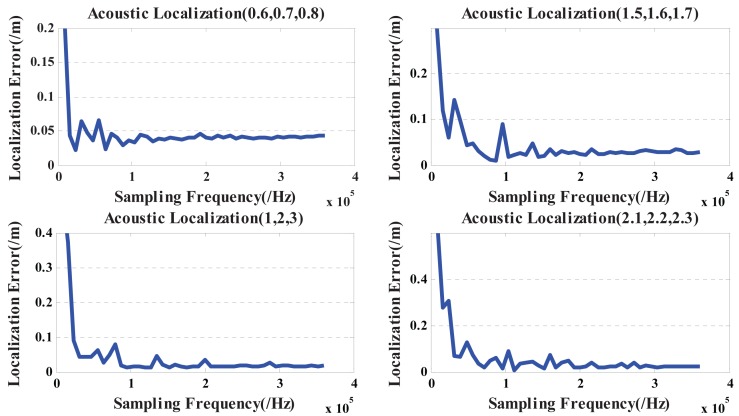
Relationship between the localization error and the sampling rate based on the GCC method.

Then, a single speech signal respectively is placed at (0.6 m, 0.7 m, 0.8 m), (1.5 m, 1.6 m, 1.7 m), (1 m, 2 m, 3 m) and (2.1 m, 2.2 m, 2.3 m). The adopted sampling frequency is from 8 kHz to 320 kHz (step size of 8 kHz), and the noise is 30 dB Gaussian noise. Therefore, localization errors based on the GCC method under the different sampling rates are given in Figure 2.

Obviously, increasing the sampling frequency *F* can reduce the error of the TDOA estimation *τ_ij_* and localization results. Thus, the above discussion demonstrates that the theorem is always tenable.

Inference 1: Localization errors rapidly decrease with the increase of the sampling rate and start to level off when the sampling rate is over 100 kHz (as shown in Figure 2). Hence, for the speech signal with the sampling rate of 8 kHz according to the G.711 standard, the minimum of the interpolation factor should be greater than or equal to 13, if the sampling rate reaches more than 100 kHz.
Inference 2: For the near-field 3D localization based on the proposed method, 15 is the optimal interpolation factor.

**Figure 3:**
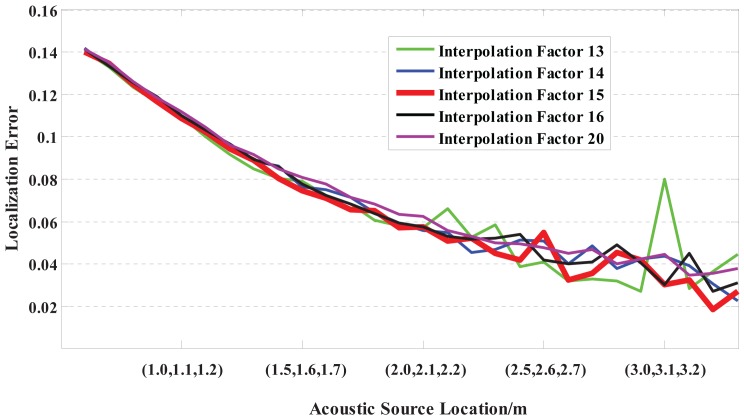
Relationship between the localization error and the interpolation factor based on the US-GCC method. Green, blue, red, black and pink curves respectively represent the localization error when the interpolation factor is set to 13, 14, 15, 16 and 20.

Firstly, for a near-field speech signal (sampling rate of 8 kHz) at coordinates ranging from (0.5 m, 0.6 m, 0.7 m) to (3.5 m, 3.6 m, 3.7 m) (step size of 0.1 m), localization error curves with different interpolation factors based on the US-GCC method are shown in Figure 3.

Apparently, the error curve change with the interpolation factor of 13 (green curve) is larger and gradually increases, and also, the localization error with the interpolation factor of 15 (red curve) is smallest compared with the other curves.

Further, the standard deviation (SD) of the acoustic source location based on the proposed method is used to select the optimal interpolation factor. In terms of statistics, the SD is defined as the uncertainty parameter, which represents the error impact on the estimated results. Namely, lower uncertainty illustrates a smaller error value range, which leads to lower error impact on the estimated results and higher estimation accuracy. Meanwhile, when estimation points are more than 10, SD should be given by Equation (11) according to Bessel formula:

(11) $$s=\sqrt{\frac{{\text{∑}}_{i=1}^{n}v_{i}^{2}}{n-1}}$$

where *n* represents the estimator number, *v* represents the difference between the true value *x_i_* and the estimated value *x′*.

When the interpolation factor respectively is set to 13, 14, 15, 16 and 20, the SD estimation via Equation (12) can be obtained substituting 25 (*n* = 25) estimation points from (0.5 m, 0.6 m, 0.7 m) to (3 m, 3.1 m, 3.2 m) (stage size of (0.1 m, 0.1 m, 0.1 m)) into Equation (11):

(12) $$\{\begin{array}{l} s_{13}\approx \sqrt{\frac{0.1483}{24}}\approx 0.07862\\ s_{14}\approx \sqrt{\frac{0.1465}{24}}\approx 0.07817\\ s_{15}\approx \sqrt{\frac{0.1419}{24}}\approx 0.07688\\ s_{16}\approx \sqrt{\frac{0.1476}{24}}\approx 0.07843\\ s_{20}\approx \sqrt{\frac{0.1542}{24}}\approx 0.08016 \end{array}$$

Obviously, the SD of the estimation result with an interpolation factor of 15 is minimum compared with the others. Hence, in this paper, 15 is selected as the optimal interpolation factor for the near-field 3D localization based on the US-GCC method.

## 3. Simulation and Experiment

To verify the feasibility and the superiority of the proposed localization algorithm in Section 2, firstly, localization results and the computation time based on the GCC method and the US-GCC method at a low sampling rate are computed via numerical simulations. Then, localization experiments have been conducted indoors based on the established simple and small portable passive acoustic source localization platform with a five-element cross microphone array (hardware size of the control part: 15.3 cm × 22.5 cm).

### 3.1. Comparison of Localization Result and Computation Time Based on the GCC Method and the US-GCC Method

**Figure 4:**
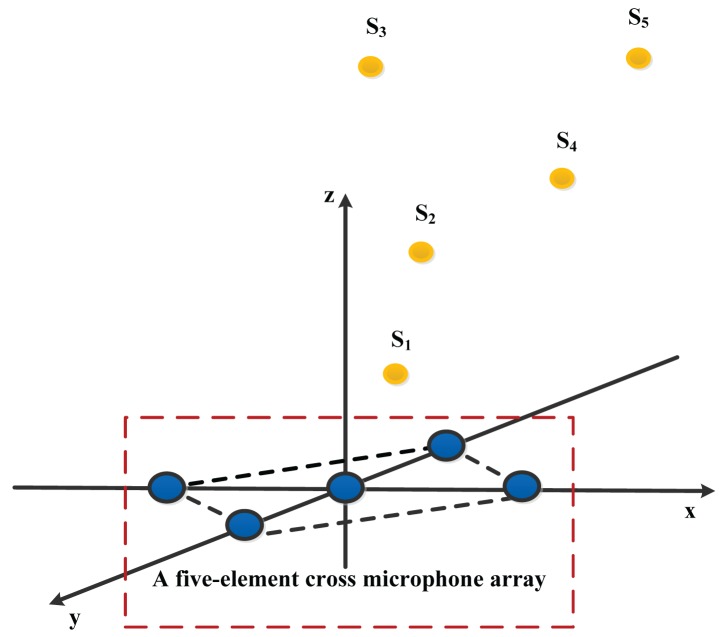
Diagram of the locations of a single speech signal for localization simulations.

In this subsection, the simulation parameters are explained as follows:

(1) Source location (as shown in Figure 4): a single speech signal recorded by the computer in a quiet environment that can be played back through a speaker. The final signal is sampled via the sampling rate of 8 kHz and assuming that it is collected by a five-element cross microphone array (see Figure 6 for its localization model). Localization simulations are repeated for five different source positions, these are: *S*_1_(0.5 m, 0.6 m, 0.7 m), *S*_2_(1.5 m, 1.6 m, 1.7 m), *S*_3_(1 m, 2 m, 3 m), *S*_4_(2.1 m, 2.2 m, 2.3 m), and *S*_5_(3 m, 3.1 m, 3.2 m).
(2) Noise model: mutually-independent white Gaussian noise is added to each microphone signal. The signal-to-noise ratio (SNR) is set to 10 dB, 20 dB and 30 dB.
(3) Interpolation factor of the US: 15.

The comparison of the simulation results based on the GCC method and the US-GCC method at a low sampling rate is described in Table 1.

Defining *e_tradition_* and *e_interp_* as the absolute error of the localization results on the GCC method and the US-GCC method, respectively, the ratio of the absolute error of both methods can be written as:

(13) $$\{\begin{array}{ll} \left(0.6,0.7,0.8\right): & \frac{e_{\text{interp}}}{e_{\text{tradition}}}\approx \frac{1}{12}\\ \left(1.5,1.6,1.7\right): & \frac{e_{\text{interp}}}{e_{\text{tradition}}}\approx \frac{1}{14}\\ \left(1,2,3\right): & \frac{e_{\text{interp}}}{e_{\text{tradition}}}\approx \frac{1}{14}\\ \left(2.1,2.2,2.3\right): & \frac{e_{\text{interp}}}{e_{\text{tradition}}}\approx \frac{1}{15}\\ \left(3,3.1,3.2\right): & \frac{e_{\text{interp}}}{e_{\text{tradition}}}\approx \frac{1}{14} \end{array}$$

Equation (13) shows that the absolute error of the localization results based on the US-GCC method is from 1/15- to 1/12-times that based on the GCC method with the same sampling rate. Therefore, the proposed method significantly improves the accuracy of the TDE and, consequently, the acoustic source location estimated at a low sampling rate.

**Figure 5:**
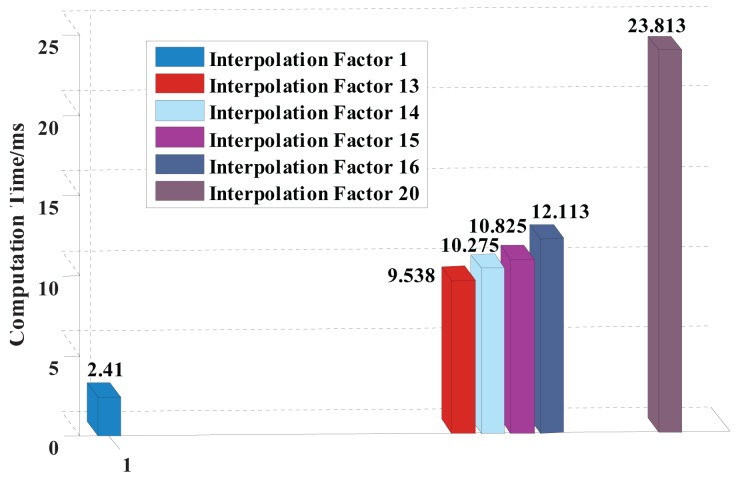
Localization computation times based on the GCC method and US-GCC method in the case with the interpolation factor of 13,14,15,16 and 20.

Next, the localization computation times (as shown in Figure 5) based on the GCC method and US-GCC method (with the different interpolation factors) are calculated in the advanced reduced instruction set computing machines (ARM7:LPC2148). The main frequency is 60 MHz, and the sampling points are 3500.

Apparently, localization computation time based on the GCC-US method with the interpolation factor of 15 is 10.825 ms and only 8.415 ms more than the 2.410 ms based on the GCC method.

### 3.2. Passive Acoustic Source Localization Platform

The hardware part of the established localization platform mainly includes a five-element cross microphone array, a signal preprocessing circuit and an MCU. The five-element cross microphone array is employed to receive the acoustic signal. After the amplifier circuit and the filter circuit, the signal then is sent to the upper PC through the MCU for software processing and showing the localization results.

#### 3.2.1. Five-Element Cross Microphone Array

##### Localization model of the five-element cross microphone array

**Figure 6:**
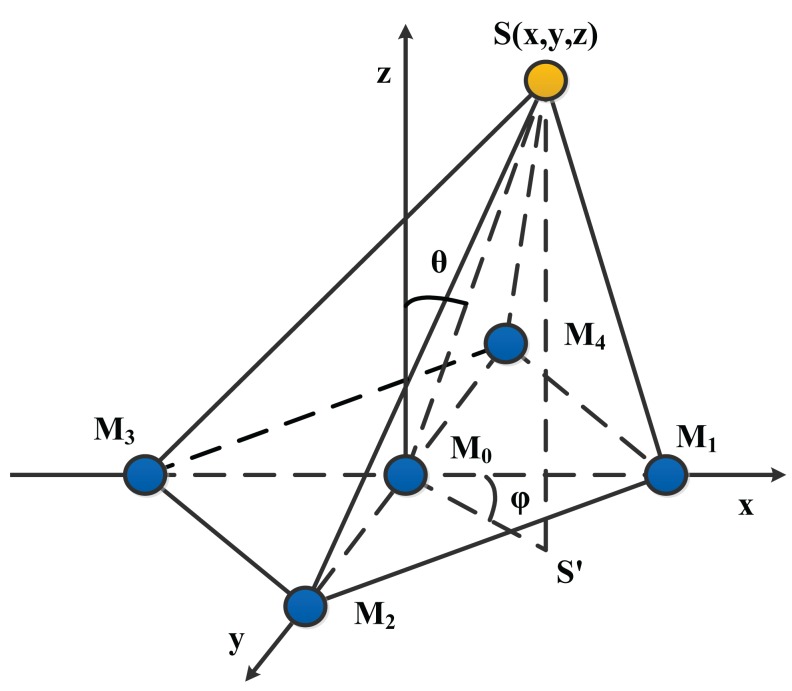
Geometrical model of the five-element cross microphone array.

The minimum number of microphones required for 3D localization is four. Yet, more microphones will increase the complexity of the localization algorithm, so in this paper, the five-element cross microphone array [13] is employed because of its higher reliability and accuracy compared with the four-element cross array. The localization model of the five-element cross array is shown in Figure 6. *S* is an acoustic source placed at the unknown coordinate (*x*, *y*, *z*). Angle *θ* from the positive *Z* axis to M_0_S is defined as the pitch angle, and angle *φ* from the positive *X* axis to M_0_S' is defined as the azimuth angle. The coordinates of the five microphones are as follows: *M*_0_(0,0,0), *M*_1_(*D*,0,0), *M*_2_(0,*D*,0), *M*_3_(−*D*,0,0), *M*_4_(0,−*D*, 0), where *D* is the known distance between microphone *M*_0_ and the others.

Considering the acoustic source as a point source and the microphone *M*_0_ as a reference point, thus according to *Distance* = *Time* × *Speed* and the geometrical model of the five-element cross microphone array, the localization equations are written as:

(14) $$\{\begin{array}{ll} x^{2}+y^{2}+z^{2} & =r^{2}\\ {\left(x-D\right)}^{2}+y^{2}+z^{2} & ={\left(r-\tau_{1}\cdot c\right)}^{2}\\ x^{2}+{\left(y-D\right)}^{2}+z^{2} & ={\left(r-\tau_{2}\cdot c\right)}^{2}\\ {\left(x+D\right)}^{2}+y^{2}+z^{2} & ={\left(r-\tau_{3}\cdot c\right)}^{2}\\ x^{2}+{\left(y+D\right)}^{2}+z^{2} & ={\left(r-\tau_{4}\cdot c\right)}^{2} \end{array}$$

where *r* is the distance between the acoustic source and the coordinate origin, *τ_i_* (*i* = 1, 2, 3, 4) is the time delay between microphone *M*_0_ and *M_i_* (*i* = 1, 2, 3, 4) and *c* is sound velocity (in this paper *c* = 340 m/s) and under the assumptions of a constant speed for an indoor experiment and a near-field source localization [4,14,15]. In addition, there is mathematical relationship between the Cartesian coordinates and the spherical coordinates obtained by Equation (15):

(15) $$\{\begin{array}{ll} x & =r\sin\theta \cos\varphi \\ y & =r\sin\theta \sin\varphi \\ z & =r\cos\varphi \end{array}$$

Therefore, for the near-field localization, the signal location estimations are calculated via substituting Equation (15) into Equation (14):

(16) $$\{\begin{array}{ll} r & =\frac{\left(c^{2}{\text{∑}}_{i=1}^{4}\tau_{i}^{2}-4D^{2}\right)}{2c{\text{∑}}_{i=1}^{4}\tau_{i}}\\ \tan\varphi & =\frac{\left(\tau_{2}-\tau_{4}\right)\left[2r-c\left(\tau_{2}+\tau_{4}\right)\right]}{\left(\tau_{1}-\tau_{3}\right)\left[2r-c\left(\tau_{1}+\tau_{3}\right)\right]}\\ \sin\theta & =\frac{{\text{∑}}_{i=1}^{4}\left\{\left[D^{2}+2rc\tau_{i}-c\tau_{i}^{2}\right]\cdot \cos\left[\varphi -\left(i-1\right)\cdot \frac{\pi }{2}\right]\right\}}{4rD} \end{array}$$

On the one hand, Equation (16) shows that signal location estimations can be obtained as long as estimating the TDOA, and a larger TDOA error will significantly decrease the localization accuracy. On the other hand, based on the above equations, the impact of the array elements' spacing and the angle on the signal location parameter accuracy is discussed and analyzed.

Taking the partial derivative of the Equation (16) with respect to the TDOA, one can obtain Equation (17):

(17) $$\frac{\partial r}{\partial \tau_{i}}\approx \frac{2rc\left(c\tau_{i}-r\right)}{D^{2}\left(\sin^{2}\theta -4\right)}\left(i=1,2,3,4\right)$$

Therefore, the relational expression of the distance variance can be written as follows:

(18) $$\sigma_{r}=\sqrt{{\left(\frac{\partial r}{\partial \tau_{1}}\right)}^{2}\sigma_{\tau }^{2}+{\left(\frac{\partial r}{\partial \tau_{2}}\right)}^{2}\sigma_{\tau }^{2}+{\left(\frac{\partial r}{\partial \tau_{3}}\right)}^{2}\sigma_{\tau }^{2}+{\left(\frac{\partial r}{\partial \tau_{4}}\right)}^{2}\sigma_{\tau }^{2}}\approx \frac{4rc\sqrt{D^{2}+r^{2}}}{D^{2}\left(4-\sin^{2}\theta \right)}\sigma_{\tau }$$

Similarly, taking the partial derivative of the Equation (16) with respect to the TDOA, the azimuth angle variance Equation (19) and the pitch angle variance Equation (20) can be written as follows:

(19) $$\{\begin{array}{l} \{\begin{array}{c} \frac{\partial \varphi }{\partial \tau_{1}}=-\frac{\partial \varphi }{\partial \tau_{3}}=\frac{\tau_{4}-\tau_{2}}{\left(1+\tan^{2}\varphi \right){\left(\tau_{3}-\tau_{1}\right)}^{2}}\\ \frac{\partial \varphi }{\partial \tau_{2}}=-\frac{\partial \varphi }{\partial \tau_{4}}=\frac{1}{\left(1+\tan^{2}\varphi \right){\left(\tau_{3}-\tau_{1}\right)}^{2}} \end{array}\\ \sigma_{\varphi }=\sqrt{{\left(\frac{\partial \varphi }{\partial \tau_{1}}\right)}^{2}\sigma_{\tau }^{2}+{\left(\frac{\partial \varphi }{\partial \tau_{2}}\right)}^{2}\sigma_{\tau }^{2}+{\left(\frac{\partial \varphi }{\partial \tau_{3}}\right)}^{2}\sigma_{\tau }^{2}+{\left(\frac{\partial \varphi }{\partial \tau_{4}}\right)}^{2}\sigma_{\tau }^{2}}=\frac{\sqrt{2}c}{D\sin\theta }\sigma_{\tau } \end{array}$$

(20) $$\{\begin{array}{l} \{\begin{array}{c} \frac{\partial \theta }{\partial \tau_{1}}=-\frac{\partial \theta }{\partial \tau_{3}}=\frac{2c^{2}\left(\tau_{1}-\tau_{3}\right)}{D^{2}\sin2\theta }\\ \frac{\partial \theta }{\partial \tau_{2}}=-\frac{\partial \theta }{\partial \tau_{4}}=\frac{2c^{2}\left(\tau_{2}-\tau_{4}\right)}{D^{2}\sin2\theta } \end{array}\\ \sigma_{\theta }=\sqrt{{\left(\frac{\partial \theta }{\partial \tau_{1}}\right)}^{2}\sigma_{\tau }^{2}+{\left(\frac{\partial \theta }{\partial \tau_{2}}\right)}^{2}\sigma_{\tau }^{2}+{\left(\frac{\partial \theta }{\partial \tau_{3}}\right)}^{2}\sigma_{\tau }^{2}+{\left(\frac{\partial \theta }{\partial \tau_{4}}\right)}^{2}\sigma_{\tau }^{2}}=\frac{2\sqrt{2}c}{D\cos\theta }\sigma_{\tau } \end{array}$$

**Figure 7:**
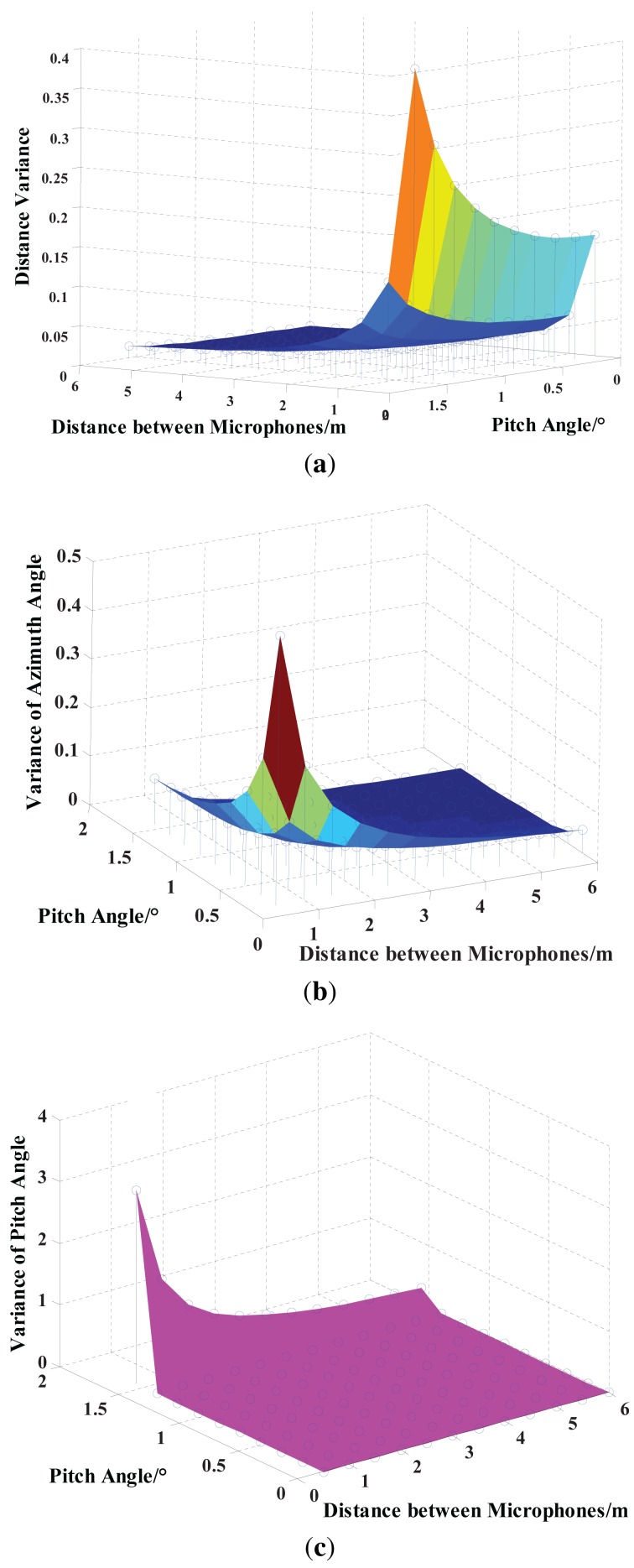
Relationship diagrams between the location parameter and its variance. (**a**) Variance diagram of the acoustic source distance from Equation (20); (**b**) variance diagram of the acoustic source azimuth angle from Equation (21); (**c**) variance diagram of the acoustic source pitch angle from Equation (22).

Obviously, besides the TDOA, the array elements' spacing *D* and signal pitch angle *θ* also have an impact on the location parameter accuracy. Therefore, assuming the constant TDOA variance (*σ_τ_* = 0.0001) in Equations (18)–(20), the relationship between the location parameter and parameter variance is discussed and shown in Figure 7.

Figure 7a demonstrates that the target distance variance increases with the increase of the pitch angle, and also, increasing the array elements' spacing can reduce distance variance. Increasing the array elements' spacing and pitch angle contributes to the decrease of the azimuth angle variance in Figure 7b. This further illustrates that the five-element cross microphone array is more advantageous to locate the azimuth angle of a low altitude target. From Figure 7c, the pitch angle variance reduces by increasing the array elements' spacing or decreasing the pitch angle.

##### Hardware design of the five-element cross microphone array

**Figure 8:**
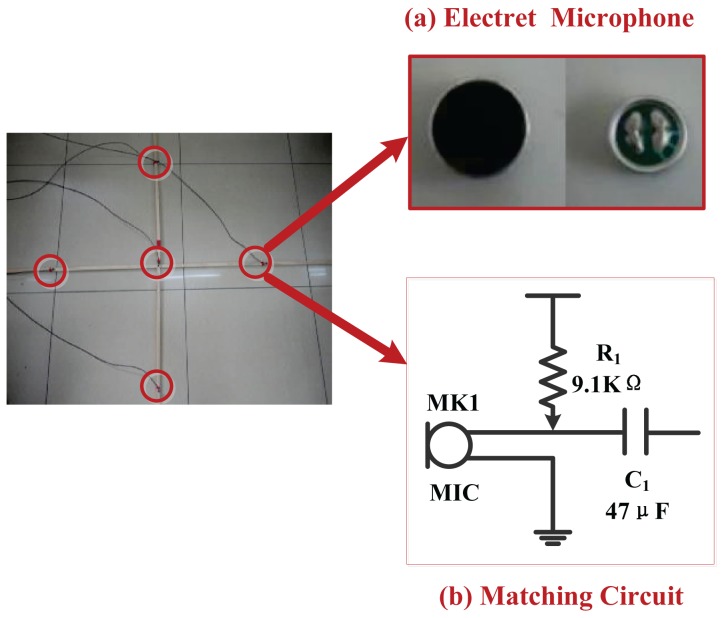
Hardware design of the five-element cross microphone array. (**a**) The electret microphone is applied to receive and convert the acoustic signal into the electric signal, as well as to amplify the converted signal through the field effect transistor (FET); (**b**) the FET completes the signal amplification as long as it works in the saturated zone that needs a matching circuit. In general, the resistance of *R*_1_ is always higher from three- to five-times the output resistance of the microphone. By testing, the output resistance of the microphone is about 2 kΩ, so the resistance of *R*_1_ is set to 9.1 kΩ.

Five electret microphones are fixed on four endpoints and a center of the 2 m × 2 m cross wooden support, respectively (as shown in Figure 8). Meanwhile, to reduce electromagnetic interference, the shielded wire is employed as the guide line that connects five microphones to the preprocessing circuit's PCB.

In this paper, the reason for using electret microphones is that they are often very inexpensive and have a simple structure, small size, are light weight, have a wide frequency response ranging from 20 Hz to 20 kHz and a small transient distortion [16].

#### 3.2.2. Signal Preprocessing Circuit

**Figure 9:**
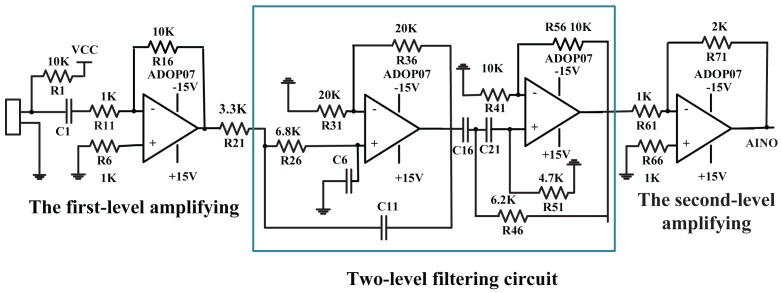
The signal preprocessing circuit includes two amplifying circuits and a two-level filtering circuit.

The signal preprocessing circuit (as shown in Figure 9) is designed to amplify and filter weak output signals from five microphones. For a speech signal with a general frequency range from 300 to 3400 Hz and a wider pass-band width, a low pass filter and a high pass filter are exploited to remove noises, and also, their cutoff frequencies are 3400 Hz and 300 Hz, respectively. Moreover, the second amplifying of the two amplifying circuits (total amplification factor: 20) is employed following the two-level filtering circuit later.

#### 3.2.3. MSP430F149 MCU

The smallest development board, the TIMSP430F149, can easily record a program because of being loaded with the RS232 communication module, reset module and power module, *etc*. Hence, it is widely applied as the core control of the signal processing. However, the MSP430F149 MCU can only complete sampling and conversion for a single signal at a time, namely it cannot achieve synchronous sampling for multiple signals. Therefore, the system collects the acoustic signal using the alternating sampling mode of the MSP430F149 MCU. Yet, there is a sampling time delay between adjacent channels that should be calculated for the TDE compensations. Defining the sampling time delay *T_S_* as:

(21) $$\{\begin{array}{ll} T_{s} & \approx T_{h}+T_{t}\\ T_{h} & =4\times \text{ADCclock}\left(\text{clk}\right)\times N\left(N=4\right)\\ T_{t} & =13\times \text{ADCclk}\times F_{\text{adc}}\\ F_{adc} & =\frac{1}{2\times 3.14\times R\times C}\left(R=2K,C=30pF\right) \end{array}$$

where *T_h_* is hold time, *T_t_* is conversion time, *ADCclk* is an ADC12clock source (8 M) and *F_adc_* is the frequency of the ADC12 equivalent circuit. Therefore, the final TDE is presented by Equation (22):

(22) $$\tau_{ij}={\hat{\tau }}_{ij}+\left(i-j\right)\cdot T_{s}\;\left(j=1,i=2,3,4,5\right)$$

where *τ^_ij_* is the TDE based on the GCC method.

#### 3.2.4. Localization Experiment and Discussion

**Figure 10:**
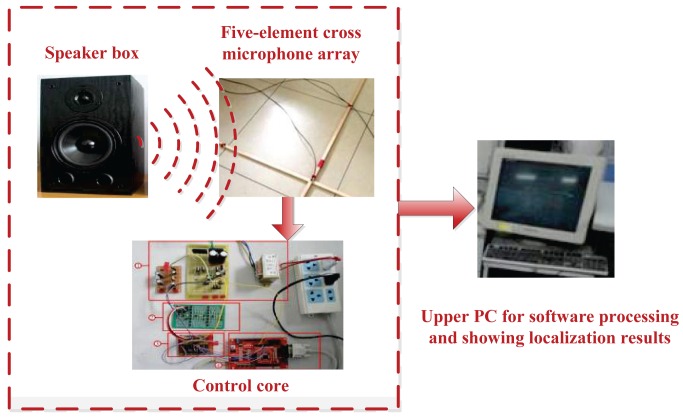
Passive acoustic source localization experimental platform with a five-element cross microphone array.

To verify the distributed acoustic source localization capabilities of the constructed localization platform under a low sampling rate, localization experiments are carried out in a room with a low reverberance (as shown in Figure 10). The room dimension is 9 m × 8 m × 3 m (*x* × *y* × *z*). Additionally, considering different environmental noise sources (from fans, PCs, lights, a few babble noises from outside, *etc*.), the noise field can be approximated as a diffuse one.

The experimental conditions are explained as follows:

(1) Array structure and location: a five-element cross microphone array with a spacing of 1 m.
(2) Acoustic source: a single speech signal from a speaker box.
(3) Sampling frequency: 8 kHz.
(4) Interpolation factor for the US operation 15.
(5) Sound velocity: 340 m/s, ignoring temperature changes indoors.

**Figure 11:**
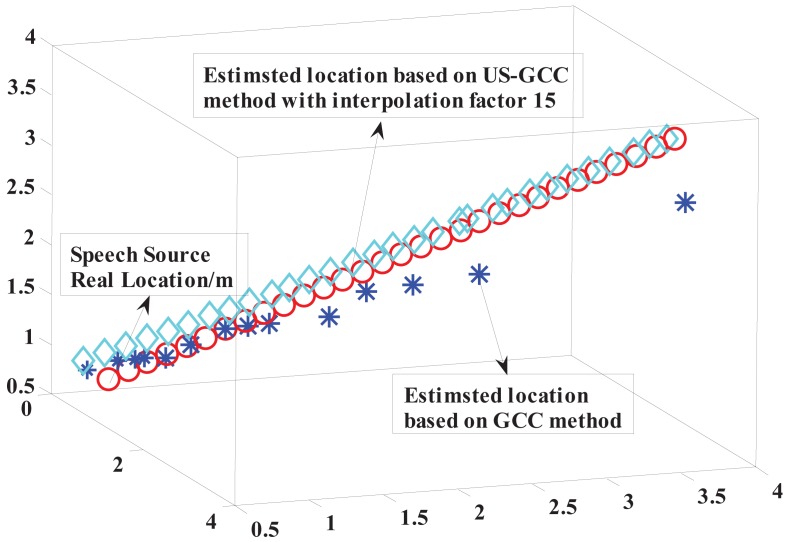
Real location and the estimated location of the acoustic source based on the GCC and the US-GCC method.

Firstly, the five-element cross microphone array receives the speech signal (emitted by the speaker box) placed at some different coordinates. Then, the received signals after amplifying and filtering are sent to the upper PC via the MCU for the subsequent localization processing based on the proposed US-GCC method and showing the localization results. At the same time, the endpoint detection of the speech signal and reverberation suppression are generally processed for the acoustic source localization platform (see Appendices A and B). Finally, experimental results (as shown in Table 2) at a low sampling rate show that relative errors of the distance *r*, the azimuth angle *φ* and the pitch angle *θ* respectively, are about 20%, 10% and 20% within a certain distance. Meanwhile, the real location and estimated location of the acoustic source (as shown in Figure 11) present that the localization accuracy based on the US-GCC method has been significantly improved, compared with that based on the GCC method.

**Figure 12:**
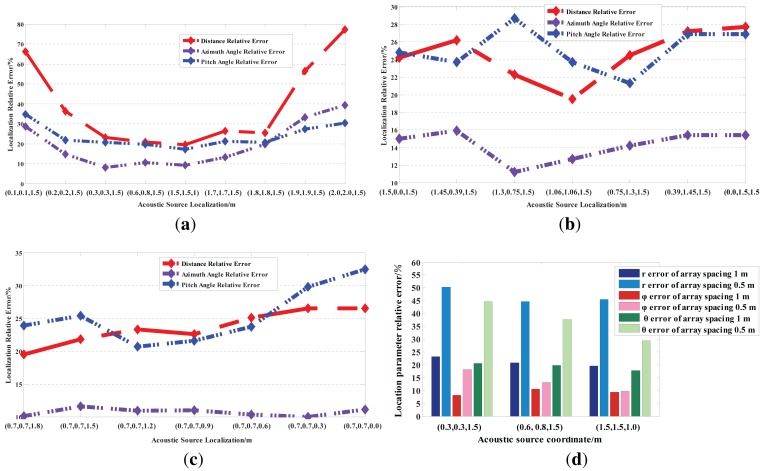
Localization performances of the established platform at a low sampling rate. (**a**) The speech signal is placed at the horizontal plane XOY , and its height remains invariable; (**b**) the speech signal is moved every other 30° on the semi-circle with a 1.5-m radius in the first quadrant of the horizontal plane XOY; (**c**) the speech signal is placed at coordinates from (0.7 m, 0.7 m, 1.8 m) to (0.7 m, 0.7 m, 0 m), namely it moves every other 0.3 cm on the *z* axis, and the coordinates of *x* and *y* remain invariable; (**d**) the localization error comparison under the array element spacing of 0.5 m and 1 m, respectively.

In addition, the localization performances of the established platform at a low sampling rate are depicted in Figure 12.

From Figure 12a, the relative errors of all location parameter are less than 30% from (0.3 m, 0.3 m, 1.5 m) to (1.8 m, 1.8 m, 1.5 m). Hence, the localization distance range approximately is from (0.32 + 0.32)^1/2^ ≈ 0.42 m to (1.82 + 1.82)^1/2^ ≈ 2.54 m at the horizontal plane XOY. In Figure 12b, the azimuth angle *φ* has a higher accuracy compared with both the distance r and the pitch angle *θ*. The relative errors of the azimuth angle *φ* are less than 20%, and the relative errors of both the distance *r* and the pitch angle *θ* are from 20% to 30%. Figure 12c demonstrates that the relative errors of the pitch angle *θ* increase with the increase of the pitch angle *θ*. When the pitch angle *θ* is less than 70°, its relative errors are *circa* 20%. Figure 12d shows that increasing the array element spacing can contribute to reducing the localization accuracy. At the same time, the above experimental results and discussions also validate the mathematical analysis for the five-element cross microphone array model.

Finally, comparisons of the experimental results between our system and the research results of [17,18] are presented in Tables 3 and 4. From Table 3, the absolute errors of the arrival angle of two constructed systems are approximate. Meanwhile, the reduction of the number of microphones in turn leads to a reduced localization accuracy [3]. Therefore, as shown in Table 4, the absolute errors of the pitch angle of our system increase by ±0.2° only within 2 m, compared with the system of [18], which uses an eight-element microphone array. In this sense, the system accuracy in this paper basically meets the 3D near-field localization requirements.

## 4. Conclusions

For a small portable system, especially for a multitasking micro-embedded system, a modification of the GCC method based on the US theory is proposed to improve the TDOA accuracy and, consequently, the localization accuracy at a low sampling rate. In addition, for the near-field localization, the localization error curve and computation time based on the US-GCC method under the different interpolation factors are given in this paper. According to the SD of the location estimation and localization computation time, the optimal interpolation factor is set to 15. The simulation results show that absolute error of the localization results based on the US-GCC method with the interpolation factor 15 is approximately from 1/15- to 1/12-times that based on the GCC method with the same sampling rate. Finally, our designed and established portable acoustic source localization platform based on the proposed method can perform accurate 3D near-field localization at a low sampling rate, and also, the possibility is given for applying the US-GCC method with an interpolation factor of 15 to a small portable system, especially a multitasking micro-embedded system.

## Figures and Tables

**Table 1:** Comparison of the simulation results based on the GCC method and the US-GCC method at a low sampling rate (8 kHz).

**Source Real Location/m**	**SNR**	**GCC**	Distance Absolute Error*/m	**US-GCC**	**Distance Absolute Error/m**
*(0.6, 0.7, 0.8)*	10	0.5476, 0.5780, 0.9110	0.7422	0.5784, 0.6841, 0.8311	0.06185
	20	0.5829, 0.6135, 0.8466	0.6868	0.5899, 0.6890, 0.8215	0.05591
	30	0.5876, 0.6728, 0.8163	0.5523	0.4854, 0.5649, 0.9636	0.05112

*(1.5, 1.6, 1.7)*	10	1.5510, 1.7343, 1.7414	0.6360	1.4981, 1.6011, 1.7112	0.04543
	20	1.5499, 1.6342, 1.8410	0.4552	1.4987, 1.5985, 1.7113	0.03251
	30	1.5399, 1.6340, 1.7407	0.5380	1.4996, 1.5989, 1.7001	0.03843

*(1, 2, 3)*	10	1.0510, 2.1343, 3.0414	0.5505	0.9981, 2.0011, 3.0112	0.03932
	20	1.0499, 2.0342, 3.1410	0.4423	0.9987, 1.9985, 3.0113	0.03159
	30	1.0399, 2.0340, 3.0407	0.4364	0.9996, 1.9989, 3.0001	0.03118

*(2.1, 2.2, 2.3)*	10	1.8400, 1.9400, 1.7702	0.7011	2.0810, 2.1810, 2.3300	0.04676
	20	1.8511, 1.9511, 1.7688	0.6873	2.0814, 2.1814, 2.3301	0.04582
	30	1.8531, 1.9531, 1.7679	0.6373	2.0816, 2.1816, 2.3266	0.04249

*(3, 3.1, 3.2)*	10	3.6502, 3.7502, 3.4101	0.9432	3.0202, 3.1202, 3.2410	0.06737
	20	3.3488, 3.6488, 3.3503	0.8742	3.0199, 3.1199, 3.2300	0.06244
	30	3.2422, 3.5422, 3.3236	0.6686	3.0108, 3.0010, 3.2212	0.04776

* $\Delta r=\sqrt{x^{2}+y^{2}+z^{2}}-\sqrt{{\left(x^{\prime }\right)}^{2}+{\left(y^{\prime }\right)}^{2}+{\left(z^{\prime }\right)}^{2}}$, where Δ*r* is the distance absolute error, (*x*, *y*, *z*) and (*x′* ,*y′* ,*z′* ) respectively are the real source location and the estimated source location based on the GCC method and the US-GCC method.

**Table 2:** Experimental results at a low sampling rate (8 kHz). (SNR 30 dB).

**Speech Source Real Location/m**		**Calculated Values**	**Experimental Values**	**Absolute Error/m**	**Relative Error**	**Estimated Location/m**
*(0.3, 0.3, 1.5)*	Distance (*r*/m)	1.5588	1.9022	0.3616	23.20%	*(0.46*, *0.45*, *1.79)*
	Pitching angle (*θ*/°)	15.79	12.53	3.25	20.60%	
	Azimuth (*φ*/°)	45.00	41.38	3.62	8.05%	

*(0.6, 0.8, 1.5)*	Distance (*r*/m)	1.8028	2.1778	0.2749	20.80%	*(0.54, 0.71, 1.56)*
	Pitching angle (*θ*/°)	33.69	27.05	3.64	19.70%	
	Azimuth (*φ*/°)	53.13	47.50	5.63	10.60%	

*(1.05, 1.05, 1.5)*	Distance (*r*/m)	2.1107	2.5222	0.4115	19.50%	*(0.98, 0.97, 1.59)*
	Pitching angle (*θ*/°)	45.29	34.56	10.73	23.70%	
	Azimuth (*φ*/°)	45.00	50.72	5.72	12.70%	

*(1.5, 1.5, 1.0)*	Distance (*r*/m)	2.3452	2.8025	0.4573	19.50%	*(1.42, 1.46, 1.19)*
	Pitching angle (*θ*/°)	54.76	53.55	11.21	17.30%	
	Azimuth (*φ*/°)	45.00	40.81	4.19	9.30%	

*(1.7, 1.7, 1.5)*	Distance (*r*/m)	2.8337	3.5790	0.7453	26.3%	*(1.64, 1.64, 1.59)*
	Pitching angle (*θ*/°)	58.04	45.68	12.36	21.3%	
	Azimuth (*φ*/°)	45.00	39.06	5.94	13.2%	

**Table 3:** Comparison of the experimental results between our system and the system of [17].

**Arrival Angle in This Paper** (°)			**Arrival Angle in [17]** (°)	

**Real Value**	**Estimation Value**	**Absolute Error**	**Real Value**	**Absolute Error**
5.39	3.51	1.88	0	0.00
33.42	36.6	3.18	30	4.09
60.83	69.96	9.13	60	0.73
87.78	90.53	2.75	90	1.34

**Table 4:** Comparison of the experimental results between our system and the system of [18].

**The Experimental Results of the Localization System in This Paper**				**The Experimental Results of the Localization System in [18]**		
	
**Real Location** (**m**)	**Real Distance** (**m**)	**Azimuth Angle Absolute** (°)	**Pitching Angle Absolute** (°)	**Real Distance** (**m**)	**Elevation** (°)	**Mean Angular Absolute** (°)
(1.7, 1.7, 1.5)	2.8337	5.94	9.36	3.0	8.0	3.0
(0.3, 0.3, 1.5)	1.5588	3.62	3.25	1.5	-13	3.1
(0.2, 0.2, 1.0)	1.0392	3.22	3.43	0.9	24	3.3

## Appendix

### Appendix A

Under a silent period of the speech signal, only complicated environmental noises are collected. Thus, to determine the silence signal or speech signal, using endpoint detection is necessary. In general, the speech signal is non-stationary, but it can be assumed stationary for short time scales (from 10 ms to 30 ms). Therefore, the speech signal is divided into overlapping frames. The frames are then windowed using an analysis window function. Relying on this characteristics, a short time energy and a short time zero crossing rate [19,20] can be used for the endpoint detection.

The short time energy [19] is defined as:

(A.1) $$E=\overset{n}{\underset{m=n-N+1}{\text{∑}}}{\left[x\left(m\right)w\left(n-m\right)\right]}^{2}\;n-N+1\leq m\leq n$$

where expression *E* represents the energy of the signal *x*(*m*), *w*(*n* − *m*) is the window function and *N* is the window length. In this paper, a Hamming window that has a window length of 20 ms is employed as the analysis window function. High energy would be classified as voice and lower energy as silence, namely setting the threshold to classify the speech as voice or silence. If the calculated signal energy is lower than the threshold, the speech is classified as silence, whereas if the energy is more than the threshold, the speech is classified as voice.

In addition, the zero crossing rate (ZCR) counts the number of zero crossings in the speech signal. Voiced segments have a low ZCR compared with unvoiced segments. The definition of the short time zero crossing rate is as follows:

(A.2) $$z=\overset{\infty }{\underset{m=-\infty }{\text{∑}}}\left|\text{sgn}\left[x\left(m\right)\right]-\text{sgn}\left[x\left(m-1\right)\right]\right|w\left(n-m\right)$$

where the *sgn* function is defined by Equation (A.3):

(A.3) $$\text{sgn}\left[x\left(n\right)\right]=\{\begin{array}{cc} 1 & x\left(n\right)\geq 0\\ -1 & x\left(n\right)<0 \end{array}$$

The ZCR is very useful for discriminating speech from noise and for determining the start and end of a speech segment. Lower energy in the ZCR would be classified as voice and high energy as silence.

## Appendix B

In the GCC method, if received signals are free of reverberation and are properly filtered, the GCC method reduces to the maximum likelihood time delay estimator [21]. However, in a typical room, there are direct and reflected speech signals, namely reverberation. The presence of reverberation in the received signals has disastrous effects on the performance of the GCC method. Considering *S*(*t*) as a single speech signal, collected signals *s*_1_(*t*) and *s*_2_(*t*) at Microphone 1 and Microphone 2 respectively become:

(B.1) $$\{\begin{array}{l} s_{1}\left(t\right)=S\left(t\right)+\eta S\left(t-\tau_{1}\right)\\ s_{2}\left(t\right)=S\left(t-\tau_{d}\right)+\eta S\left(t-\tau_{2}\right) \end{array}$$

where *τ_d_* is the time delay of the direct signal between microphones, *τ*_1_ is the time difference of arrival between the reflected signal and the directed signal arriving at Microphone 1, *τ*_2_ is the time difference of arrival between the reflected signal arriving at Microphone 2 and the directed signal arriving at Microphone 1 and *η* is the amplitude ratio of the reflected speech signal to the direct speech signal. The cross-correlation function of *s*_1_(*t*) and *s*_2_(*t*) can be defined as:

(B.2) $$R_{s_{1}s_{2}}\left(\tau \right)=\eta \delta \left(u-\tau_{2}\right)+\eta^{2}\delta \left(u-\left(\tau_{1}-\tau_{2}\right)\right)+\delta \left(u-\tau_{d}\right)+\eta \delta \left(u-\left(\tau_{1}-\tau_{d}\right)\right)$$

where *δ*(*u*) is the Dirac delta function. According to the characteristics of the GCC method, from Equation (B.2), it is clear that the cross-correlation function has four peaks when *u* = *τ*_2_, *u* = *τ*_1_ − *τ*_2_, *u* = *τ*_d_, *u* = *τ*_1_ − *τ_d_*, leading to it not being able to determine the correct delay time. Thus, it is necessary to remove the reverberation. In this paper, the cepstral prefiltering technique [21] is applied on the received signals before the TDOA estimation in a typical reverberant environment.

The cepstrum is defined as the inverse Fourier transform of the log-spectrum of a stationary random process [22], where the cepstrum of a discrete-time signal *x*[*n*] is given by:

(B.3) $$\hat{x}\left[k\right]=F^{-1}\left\{\text{lg}X\left(\omega \right)\right\}$$

where *X*(*ω*) is the Fourier transform of *x*[*n*], *F*^−1^{·} represents the inverse Fourier transform, the log operator is the complex logarithm and the integer variable *k* is called quefrency [21]. In the complex cepstrum domain, the complex cepstrum of the speech signal is near the origin; however, the complex cepstrum of the reverberation signal is far away from the origin. Based on this characteristic, cepstral prefiltering can be adopted to deal with the reverberation.
